# Solitary intraosseous neurofibroma of the oral cavity: rare localization in the maxilla

**DOI:** 10.1186/s12903-024-04470-9

**Published:** 2024-06-22

**Authors:** Longmei Guo, Chunling Wu, Xueyi Liang, Jiusong Han

**Affiliations:** 1 Stomatological Hospital, School of Stomatology, Southern Medical University, Guangzhou, 510280 China; 2 Department of Periodontics, Stomatological Hospital, Southern Medical University, 366 South of Jiangnan Road, Guangzhou, 510280 China; 3 Department of Oral and Maxillofacial Surgery, Stomatological Hospital, Southern Medical University, 366 South of Jiangnan Road, Guangzhou, 510280 China

**Keywords:** Solitary neurofibroma, Intraosseous, Maxilla, Palatal lesion, Immunohistochemistry

## Abstract

**Background:**

Neurofibroma is a common benign tumor of neuronal origin that can occur as a solitary tumor or as a component of the generalized syndrome of neurofibromatosis. Neurofibromas are primarily located in the subcutaneous soft tissues and commonly involve extra-oral sites. Solitary intraosseous neurofibromas of the oral cavity are infrequent, with occurrences in the maxilla being exceedingly rare.

**Case presentation:**

A 22-year-old male patient presented with an asymptomatic mass in the maxilla. Cone-beam computed tomography revealed a round, well-outlined, radiolucent lesion with expansive growth. The neoplasm with the complete capsule was completely removed and confirmed as a neurofibroma based on histopathological and immunohistochemical findings. The reported cases of solitary intraosseous neurofibromas located in the maxilla published in the English literature were compiled to assist in the diagnosis of solitary intraosseous neurofibromas of the maxilla. Nine months after the surgery, there were no signs of tumor recurrence or malignant transformation.

**Conclusions:**

This report emphasizes that rare locations of neurofibromas, such as solitary intraosseous neurofibromas in the maxilla, typically demonstrate nonspecific clinical and radiological features. Clinicians should consider solitary intraosseous neurofibromas as possible differential diagnoses and recognize the histopathological and immunohistochemical features to confirm the correct diagnosis. A longer follow-up period is required because of the potential for local recurrence and malignant transformation of these tumors.

## Background

Neurofibromas are among the most common nerve neoplasms, representing approximately 5% of all benign soft-tissue tumors [1]. Neurofibromas are typically multiple lesions associated with neurofibromatosis, generally known as neurofibromatosis type-1 (NF-1) or von Recklinghausen disease (VRD), which induces skin changes and bone deformations [2]. Neurofibromas also present as a single entity and do not exhibit other manifestations of neurofibromatosis [2, 3]. Neurofibroma, as a component of NF-1, is an autosomal dominant genetically inherited disease that causes multiple tumors. However, the exact cause of solitary neurofibroma remains unknown [2, 4]. Neurofibromas most commonly occur on the skin, and the frequency of all neurofibromas occurring in the head and neck region are reported to be approximately 25%. Solitary presentation in the oral cavity or as part of a systemic syndrome was detected in 6.5% of cases [2, 5]. Intraoral isolated neurofibromas are uncommon; they can be intraosseous, although most are extraosseous. Intraosseous neurogenic tumors of the oral cavity are rare and have a marked predilection for the posterior mandible [6]. The maxilla is an unusual site for these neoplasms. Since Toth et al. first described solitary intraosseous neurofibromas of the maxilla in 1975, only a few cases have been reported in English literature [2–8]. Herein, we report a rare case of an isolated intraosseous neurofibroma of the hard palate in the absence of syndromic neurofibromatosis, which was fortuitously discovered during systematic clinical examination.

## Case presentation

A 22-year-old male visited a local dental clinic due to left maxillary tooth pain when chewing. Dental examination revealed chronic apical periodontitis related to a carious lesion in the upper left first molar (tooth 26). In addition, swelling of the hard palatal mucosa was observed. Dental treatment consisted of 26 endodontic treatments, and the patient was referred to our department for further investigation. He described the swelling as having no obvious enlargement and reported no specific medical, family, or psychosocial history.

During the physical examination, the patient was observed to be moderately built and nourished, with no abnormalities on his body or face. Furthermore, no other signs or symptoms suggested systemic involvement.

**Fig. 1:**
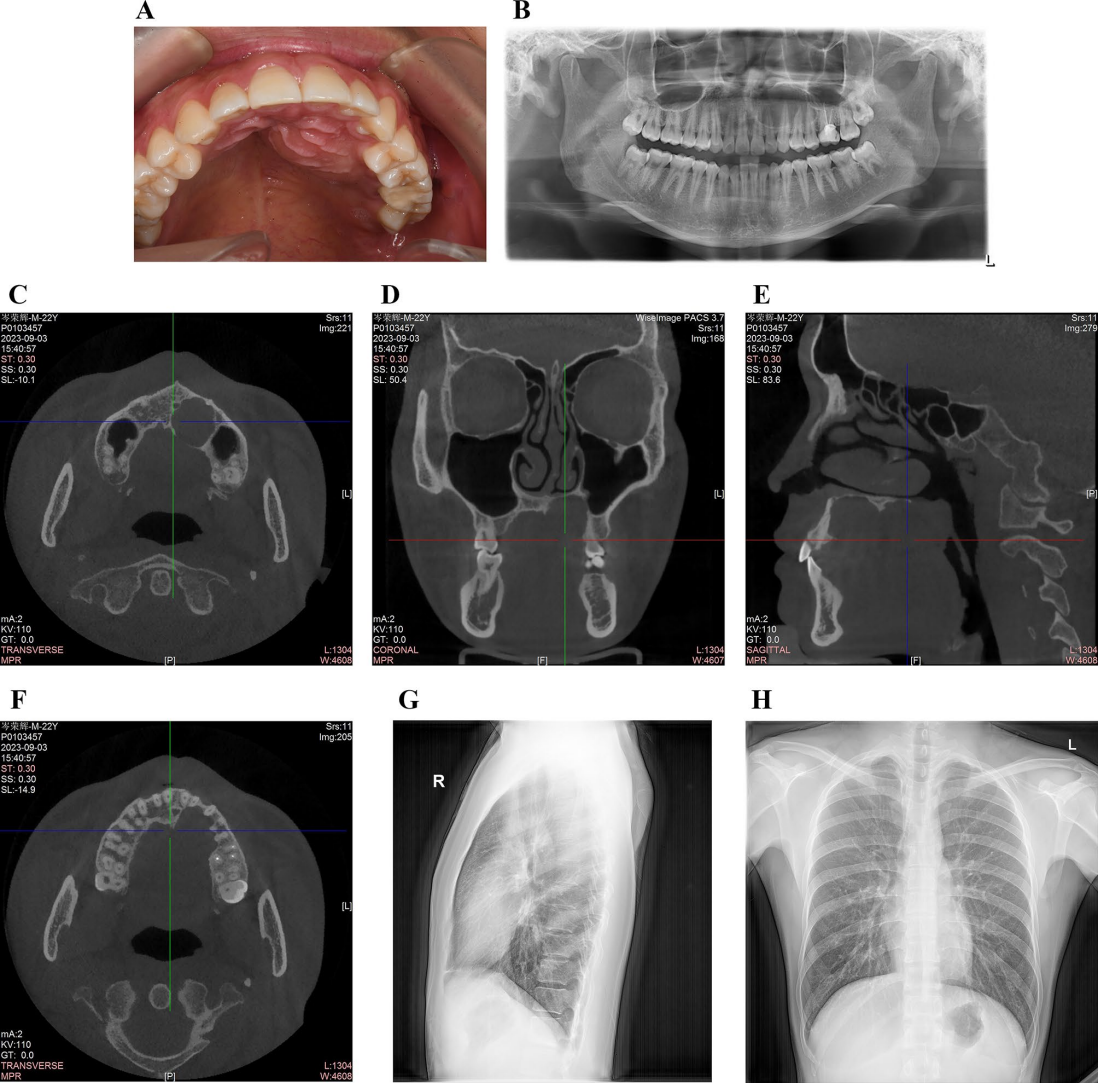
Preoperative examinations. **A**. An intraoral photograph presenting a swelling in the left of hard palate. **B**. OPG demonstrating a well-defined unilocular radiolucency with a thin radiopaque border in the left of maxilla. **C**, **D**, **E**, and **F**. CBCT images showing an expansile radiolucent intraosseous lesion with perforation of the palatal lamina and partial loss of the floor of maxillary sinus, but no obvious absorption or displacement of the adjacent tooth roots. **G** and **H**. Chest radiographs showed no abnormality

Oral examination revealed an oval swelling measuring approximately 19 mm × 22 mm on the left side of the hard palate (Fig. 1A). The overlying mucosa appeared normal. Upon palpation, the mass felt smooth, well-defined, non-tender, and soft in consistency, with no increase in local temperature. The hard palatal swelling extended from the left maxillary incisor (tooth 22) to the mesial aspect of tooth 26. No carious lesions, noticeable mobility, or discoloration was observed on the left maxillary teeth. However, the occlusal surface of tooth 26 was filled with resin. Pulp vitality testing using cold ice elicited a negative response in tooth 26 and a normal response in the other teeth. Discomfort was noted in tooth 26 during percussion. No significant bulging was observed in the buccal region of the left maxillary bone. Periodontal probing findings were within normal limits for both teeth, and the gingiva appeared intact.

The patient underwent radiographic examination. Orthopantomography (OPG) revealed a well-circumscribed homogeneous unilocular radiolucency with a thin radiopaque border on its contours in the corresponding area of the left maxilla (Fig. 1B). Cone-beam computed tomography (CBCT) images depicted a round, well-outlined radiolucent lesion (Fig. 1C), which extended medially up to tooth 21, distally to tooth 26, and up to the nasal fossa, with partial loss of the floor of the maxillary sinus (Fig. 1D). The lesion showed expansive growth, resulting in perforation of the palatal lamina and direct contact with the roots of the lateral incisor, canine, and first and second premolars. However, there were no visible signs of resorption or displacement of the roots (Fig. 1E, F). In addition, CBCT revealed that tooth 26 had undergone endodontic treatment, with radio-opaque obturation material present in the root canal space (Fig. 1F). Chest radiography revealed no abnormalities (Fig. 1G, H).

The patient was subsequently subjected to urinalysis, blood biochemical, and routine blood investigations, all of which were within normal limits.

Based on the patient history, clinical examination, and radiographic findings, odontogenic cysts, odontogenic keratocysts, and benign soft tissue tumors were included in the panel of diagnostic hypotheses. The definitive diagnosis was based on the histological and immunohistochemical findings.

**Fig. 2:**
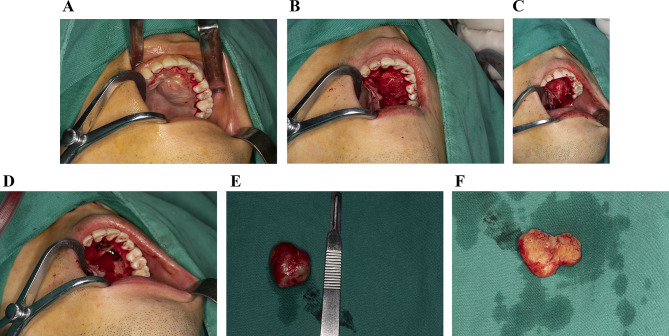
Intraoperative photographs. **A**. Surgical incision of the mass. **B**. Preparation of a mucoperiosteal fap of the lesion. **C**. Enucleation of the tumor. **D**. Partial bone loss of the maxillary sinus. **E**. Gross view of the excised specimen, showing a soft and well-demarcated mass with complete capsule. **F**. The cut surface of the mass appeared solid, homogenous, and light yellow

The planned surgical treatment consisted of the complete removal of the neoplasm followed by histopathological examination. In anticipation of intraoperative findings suggesting malignancy, excision of the lesion along with the adjacent healthy tissue and intraoperative frozen section examination were recommended. Under general anesthesia, the tumor was easily dissected from neighboring tissues owing to the presence of an intact capsule and no invasion of adjacent structures. The retrieved specimen was a well-defined, oval, soft tissue mass measuring 18 × 20 mm. A light-yellow homogenous solid was observed on the cut surface of the axial cross section of the excised mass (Fig. 2).

**Fig. 3:**
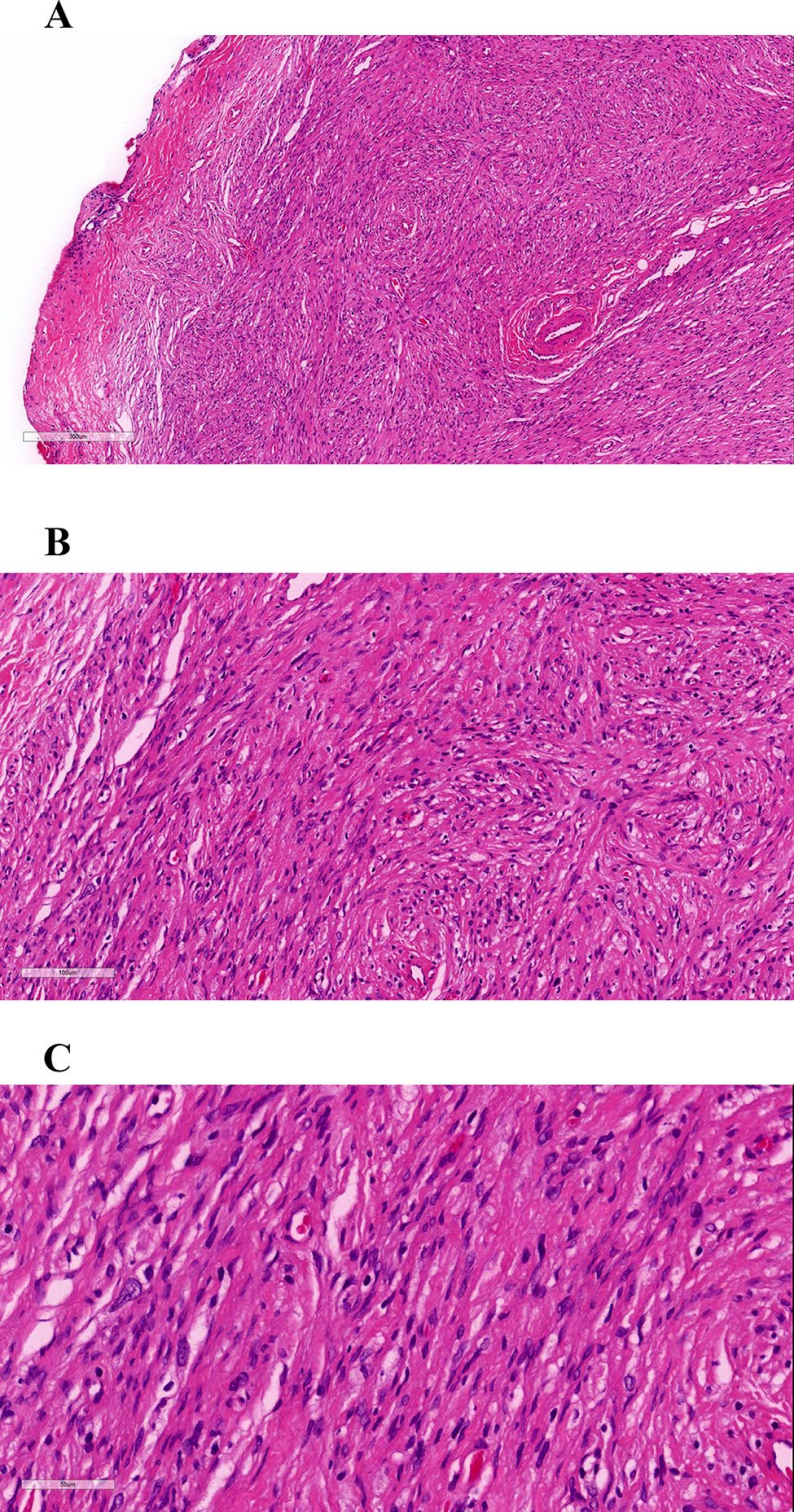
Microscopical findings. Hematoxylin and eosin stained revealed that soft tissue section, exhibiting encapsulation (**A**, ×8), consisted of spindle-shaped tumor cells with poorly defined cytoplasm and wavy nuclei (**B**, ×20, **C**, ×40)

Hematoxylin and eosin-stained soft tissue sections revealed intact encapsulation (Fig. 3A) consisting of spindle-shaped tumor cells with a poorly defined cytoplasm, wavy nuclei, and a few heterotypic nuclei (Fig. 3B, C). Immunohistochemistry was positive for S-100, CD34, SOX-10, and BCL-2, and negative for SMA, ERG, Calponin, Desmin, CD117, CD68. In addition, immunostaining for the nuclear proliferation marker Ki-67 revealed approximately 1% positivity in the tumor cells. A diagnosis of neurofibroma of the hard palate was confirmed based on these findings.

Subsequently, the patient was meticulously examined for other stigmata associated with VRD. Ophthalmological examination revealed no Lisch nodules on the iris. Pure tone and impedance audiometry results appeared normal.

**Fig. 4:**
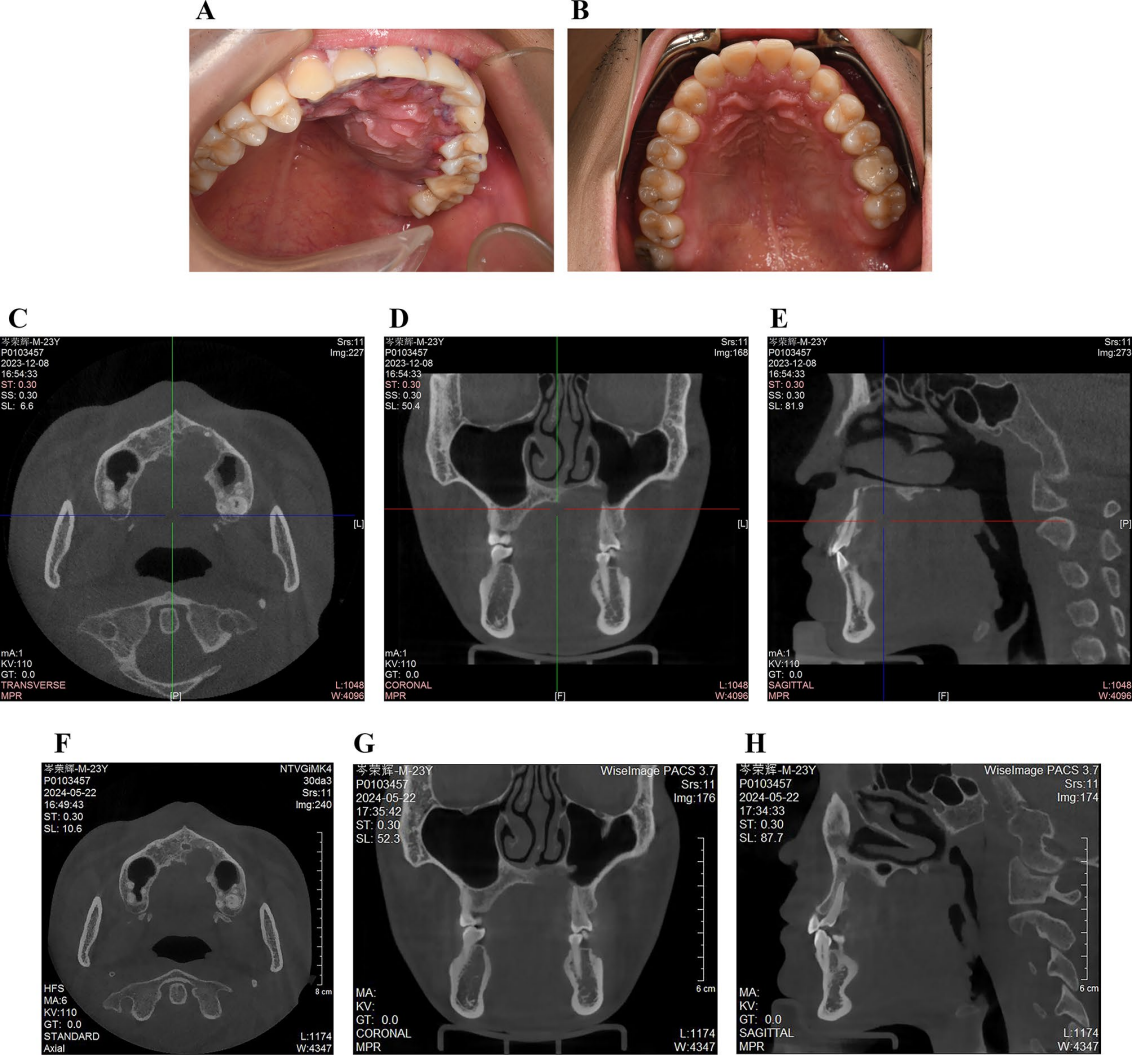
Postoperative data. **A**. An intraoral photograph presenting the swelling in operative area after one week postoperatively. **B**. Normal findings in the clinical presentation after 3 months. **C, D**, and **E**. The CBCT images showed some amount of bone formation with no sign of recurrence after 3-month follow-up. **F, G**, and **H**. The 9-month follow-up CBCT images indicated no evidence of recurrence

One week postoperatively, the wound had healed, although swelling persisted in the operative area, accompanied by slight redness of the marginal gingiva (Fig. 4A). Three months after surgery, the prognosis was fair, and the patient was asymptomatic at the operation site (Fig. 4B). The patient underwent review and was referred for a follow-up CBCT, which showed some bone formation and no signs of local recurrence (Fig. 4C, D, and E). Pulp vitality testing showed a negative response only for tooth 26, with normal responses observed for the other teeth. The patient was kept under observation for a period of nearly 9 months which presented no recurrence (Fig. 4F, G, and H).

## Discussion and conclusions

Neurofibromas are common benign tumors that originate from Schwann and mesenchymal cells that constitute the nerve sheath [2, 3, 8]. The head and neck are commonly involved because of the rich innervation of this area [7, 9]. Neurofibromas of the oral cavity often affect the trigeminal and upper cervical nerves, most of which appear in superficial soft tissues such as the tongue, buccal mucosa, gingiva, salivary glands, and palatal mucosa [2, 3, 8, 9]. Neurofibromas occurring in the bone are rare because the bone marrow space lacks nerve sheaths or myelinated nerves [6–10]. Oral intraosseous neurofibromas primarily originate from the superficial mucosa, while involvement of deeper locations resulting in bone absorption is commonly associated with subperiosteal neurofibromas [10–12]. Only a few cases of solitary intraosseous neurofibromas of the oral cavity have been described in the literature, with those in the mandible being the most common location owing to the presence of a major nerve bundle, namely, the inferior alveolar nerve [6–12]. This relative rarity of solitary intraosseous neurofibromas of the maxilla is exemplified in a review by Sharma et al., wherein of the 22 cases in their series of solitary neurofibromas involving the jawbone over the last two decades, only four were neurofibromas of the maxilla [13]. Therefore, solitary intraosseous neurofibromas of the hard palate are extremely rare, as was observed in the present case. Given its rarity, clinicians may not be familiar with intraosseous neurofibromas and therefore fail to consider it as a differential diagnosis. Herein, the reported cases of solitary intraosseous neurofibromas located in the maxilla published in the English literature until 2023 [7, 12, 14–20] were compiled to assist in diagnosis. The information presented in Table 1 (at the end of the document text file) is extracted from these cases.

**Table 1:** Summary of reports of solitary intraosseous neurofibromas in the maxilla

Reference	Age/ Gender	Symptoms	Clinical Examination	Radiographic Features	Microscopical Features	Surgical approach	Prognosis
Toth, (1975) [7]	28y/ Male	Eating difficulties	A fungating tumorous mass in the area of the tooth 26, with displaced tooth	Well-defined radiolucency, displacement of adjacent tooth	Concentric lamellar arrangements composed of spindle cells	Excision	No recurrence observed 6 months post-surgery
Brady, (1982) [14]	20y/ Female	Swelling, pain on chewing and the left naris was occluded	A large, exophytic mass extending from the maxillary tuberosity to the premolar area	An extensive, spherical, well-defined, uniformly radiolucent lesion	Spindle-shaped cell and a myxomatous network of collagen and reticulin fibers	Left radical maxillectomy	No recurrence observed 1 year post-surgery
Skouteris, (1988) [15]	16y/ Female	Painless	Firm mass in the left posterior maxillary vestibule with impacted tooth	Ill-defined radiolucency	Spindle cells and abundant myxomatous stroma	Dissection, together with the contained teeth	No recurrence observed 10 months post-surgery
Mori, (1993) [12]	18y/ Female	Painless	Gingival swelling in left maxillary premolar region, with movable and displaced teeth	Well-defined multilocular radiolucency, the roots of adjacent teeth were displaced	Wavy growth of tumor cells in a myxomatous matrix. S-100 Positive	Enucleated, together with the affected teeth	Not reported
Poupard, (1997) [16]	14y/ Male	Painless	A firm mass in the right palate	Poorly defined radiolucency, resorption of adjacent tooth	Spindle and stellate cells with a mucoid extracellular material with some fibrous tissue. S-100 Positive	Excised with a 3-mm margin of surrounding tissue	No recurrence observed 1 year after surgery
Sharma, (2009) [17]	5-month/ Male	Painless	Firm swelling on maxillary anterior right alveolar ridge	A ill-defined, homogenous iso- to hypodense mass	Densely packed collagen bundles intermingled with nerve bundles showing spindly nuclei; S-100 positive, EMA negative	Excision of the lesion along with the adjacent tissue	No recurrence observed 1year after surgery
Gogri, (2014) [18]	8y/ Female	An enlarging asymptomatic swelling	A well-defined, nodular swelling involving the maxillary anterior alveolus region	Radiolucent lesion extending from 21 to 55 and up to the nasal cavity	Tadpole, star-shaped, and spindle cells, and a wavy arrangement of fiber bundles	Excision completely	Not reported
Grewal, (2020) [19]	45y/ Male	Swelling	Hard mass in the buccal region of tooth 12, 13, 14 and 15	Slight fuzziness in the trabecular pattern in the region of tooth 13, 14 and 15	Spindle shaped cells with wavy hyperchromatic nuclei and scanty cytoplasm; S-100 positive	Excision of the lesion	Not reported
Reddy, (2020) [20]	50y/ Male	Pain and swelling	A firm diffuse swelling in relation to 11 and 21 in the midline	A welldefined oval expansile lytic lesion, resorption and migration of roots of involved teeth	Bundles of nerve fibers with wavy nuclei and densely arranged collagen fibers with inflammatory cell; S-100 and CD34 positive	Complete excision of premaxillary segment	Not reported

Only nine cases of solitary intraosseous neurofibroma in the maxilla have been previously reported, with the patients having an average age of 22.2 years. Similar to most cases that show a slight male predominance, the present case was male. Clinically, solitary neurofibromas typically occur as asymptomatic painless lesions in the initial stages. The current patient was asymptomatic and did not show any pain or neurological disturbances, similar to previously reported cases [12, 15–19]. On radiography, cortical expansion, cortical perforation, tooth displacement, and root resorption are observed in most cases [7, 12, 14–16, 20]. Cortical expansion and perforation were observed in the present case; however, displacement of the tooth or root resorption was not observed. Some studies have reported a well-defined unilocular radiographic appearance of the lesion, which was also observed in the current case [7, 14, 20].

However, a definitive diagnosis is challenging for clinicians because of nonspecific clinical and radiographic symptoms and the variety of differential diagnoses, including odontogenic cysts, odontogenic keratocysts, unicystic ameloblastoma, vascular anomalies, salivary gland tumors and other benign soft tissue tumors. Odontogenic cysts, which commonly occur as periapical cysts, are associated with infected pulpal tissues. Odontogenic keratocysts, often seen in the mandible, exhibit severe resorption of the adjacent tooth roots.

Unicystic ameloblastoma is a variant of ameloblastoma that presents as a cyst and shares clinical and radiological features with odontogenic cysts. They appear as well-circumscribed unilocular radiolucencies that often surround the crown of an impacted tooth [10]. In the present case, the teeth that were in direct contact with the neoplasm did not exhibit pulpitis, remarkable root resorption, impaction or other specific abnormalities, which is not in favor of an odontogenic cyst, odontogenic keratocyst or unicystic ameloblastoma. Vascular anomalies involving the facial skeleton are relatively uncommon. They often exhibit characteristic features, such as a sunburst, radiating spoke wheel, or reticular or soap bubble appearance in radiology [21]. Besides, a previous study indicated that the imaging characteristics of diffuse and plexiform neurofibromas can be easily confused with those of vascular anomalies, potentially leading to an incorrect diagnosis [22]. Three types of neurofibroma have been described: localized, diffuse, and plexiform [2, 22]. The solitary neurofibroma analyzed in this study appeared as a localized mass of hard palate with well-defined borders, which is not in favor of vascular anomalies. However, the possibility of salivary glands and other benign soft tissue tumors cannot be excluded. Ultrasound and magnetic resonance imaging (MRI) are valuable tools for identifying a soft-tissue mass, classifying lesions and determining the extent of the lesions [22]. Therefore, the use of MRI or oral ultrasound can help diagnose this solitary neurofibroma. In cases of persistent uncertainty, local organizational biopsy or fine-needle aspiration cytology is preferable to achieve an accurate preoperative diagnosis, with a definitive diagnosis relying on histopathological and immunohistochemical findings [12, 19]. Preoperative biopsy or fine-needle aspiration cytology were not performed for this patient since the present neoplasm appeared more likely to be benign based on preoperative clinical examination and radiographic findings.

In the present case, the lesion was successfully removed without any obvious complications. Complete surgical excision of this lesion could mainly be attributed to the presence of a complete capsule, which indicates that setting the resection margin is easier compared with most solitary non-encapsulated neurofibromas [3–6, 10, 13, 14]. Previous studies have reported that gross specimens of neurofibroma tissue appear to have a doughy consistency with a whitish and shiny surface [4, 19], which were observed in the current case. Microscopically, spindle-shaped cells with elongated, thin nuclei and scant cytoplasm surrounded by a collagenous matrix were appeared in this specimen sections. There was no salivary gland or salivary canal. These microscopic examinations can be excluded from many other diseases, especially salivary gland tumors, but differential diagnosis remains difficult to make with some soft tumors such as schwannomas. In addition to above cell arrangements, immunohistochemical findings revealed spindle-shaped cells were positive for S-100 and CD34. The S-100 protein is a useful marker for indicating a neural origin tumor [3, 4, 12]. CD34 located in the cell membrane and cytoplasm is expressed in neurofibromas but not schwannomas [3, 10].

The clinical behavior of neurofibromas is characterized by a benign course with a low frequency of recurrence after surgical excision, primarily because of the absence of a complete capsule [2–6, 23]. The local recurrence rate of this condition may be lower due to the appearance of an intact envelope. Furthermore, the risk of malignant transformation of neurofibromas is between 5 and 10%, especially for NF-1 [2, 3, 6, 10, 18]. In the present case, the tumor was an isolated intraosseous entity that was not associated with any systemic pathology and seldom changed into a malignant form. However, it is important to consider that solitary intraosseous neurofibroma may be an initial manifestation of NF-1 [8, 12] with no family history, but it can be caused by a spontaneous mutation [23, 24]. There are no distinctive features between solitary and multiple forms apart from systemic and hereditary factors; if possible, genetic studies to rule out common autosomal genetic disorders are recommended [19, 24]. Fortunately, Ki-67 staining, indicating the potential for aggressiveness and malignant transformation [3, 25], was found to be weak upon immunohistochemical analysis, and it showed no clinical features of invasion owing to the presence of an intact capsule. However, sufficient follow-up and tracking is required. We conducted clinical follow-up for the past 9 months; there were no signs of malignant transformation, recurrence or clinical manifestations of NF-1 in the present patient.

In conclusion, the present case of a single neurofibroma involving the maxillary bone with a complete envelope is extremely uncommon. The preoperative diagnosis of a solitary intraosseous neurofibroma is challenging because of its rare location and nonspecific radiographic and clinical characteristics. Histopathological analyses supported by immunohistochemistry are essential for the correct diagnosis of these rare entities. Genetic studies are required to rule out genetically inherited diseases, when possible. A longer follow-up period is required because of the potential for local recurrence and malignant transformation of these tumors.

## Data Availability

The datasets used and/or analysed during the current study are available from the corresponding author on reasonable request.
